# Senolytic effects of exercise in human muscles require acute inflammation

**DOI:** 10.18632/aging.205827

**Published:** 2024-05-15

**Authors:** Wei-Horng Jean, Yin-Chou Lin, Pei-Yao Ang, Kazushige Goto, Chao-An Lin, Luthfia Dewi, Yu-Chieh Liao, Chih-Yang Huang, Chia-Hua Kuo

**Affiliations:** 1 Department of Anesthesiology, Far East Memorial Hospital, New Taipei City 220, Taiwan; 2 Department of Physical Medicine and Rehabilitation, Chang Gung Memorial Hospital, Taoyuan 33378, Taiwan; 3 Laboratory of Exercise Biochemistry, University of Taipei, New Taipei City 11153, Taiwan; 4 Faculty of Sport and Health Science, Ritsumeikan University, Kusatsu 525-8577, Japan; 5 Cardiovascular and Mitochondrial Related Disease Research Center, Hualien Tzu Chi Hospital, Buddhist Tzu Chi Medical Foundation, Hualien 970, Taiwan; 6 Department of Medical Research, China Medical University Hospital, China Medical University, Taichung 404, Taiwan; 7 Graduate Institute of Biomedical Sciences, China Medical University, Taichung 404, Taiwan; 8 Department of Health Management and Enhancement, Open University of Kaohsiung, Kaohsiung 812, Taiwan

**Keywords:** p16^INK4a^, high-intensity intermittent exercise, cellular senescence, inflammation, NSAID

## Abstract

Higher intensity exercise, despite causing more tissue damage, improved aging conditions. We previously observed decreased p16^INK4a^ mRNA in human skeletal muscle after high-intensity interval exercise (HIIE), with no change following equivalent work in moderate-intensity continuous exercise. This raises the question of whether the observed senolytic effect of exercise is mediated by inflammation, an immune response induced by muscle damage. In this study, inflammation was blocked using a multiple dose of ibuprofen (total dose: 1200 mg), a commonly consumed nonsteroidal anti-inflammatory drug (NSAID), in a placebo-controlled, counterbalanced crossover trial. Twelve men aged 20–26 consumed ibuprofen or placebo before and after HIIE at 120% maximum aerobic power. Multiple muscle biopsies were taken for tissue analysis before and after HIIE. p16^INK4a+^ cells were located surrounding myofibers in muscle tissues. The maximum decrease in p16^INK4a^ mRNA levels within muscle tissues occurred at 3 h post-exercise (−82%, *p* < 0.01), gradually recovering over the next 3–24 h. A concurrent reduction pattern in CD11b mRNA (−87%, *p* < 0.01) was also found within the same time frame. Ibuprofen treatment attenuated the post-exercise reduction in both p16^INK4a^ mRNA and CD11b mRNA. The strong correlation (r = 0.88, *p* < 0.01) between p16^INK4a^ mRNA and CD11b mRNA in muscle tissues suggests a connection between the markers of tissue aging and pro-inflammatory myeloid differentiation. In conclusion, our results suggest that the senolytic effect of high-intensity exercise on human skeletal muscle is mediated by acute inflammation.

## INTRODUCTION

Most cells in the human body have a short lifespan, and senescent cells are detectable in a wide range of tissues from a very early age, well before reaching young adulthood [1]. p16^INK4a^ mRNA is a widely used biomarker to measure cellular senescence [2–4], and is expressed in replicable cells (i.e., stem cells). Elevated p16^INK4a^ expression in tissues inhibits cell proliferation, delays wound healing, and impairs physical fitness [5]. Clearance of senescent cells has been shown to enhance physical fitness and healthspan in animals [6]. Senescent cell accumulation in tissues also results in low-grade chronic inflammation [7].

Cellular senescence has been shown to increase pro-inflammatory myeloid differentiation for phagocytosis [8, 9], characterized by increased CD11b mRNA of myeloid cells in tissues [10, 11]. Inflammation is a local immune response that functions to recognize and clear senescent cells [12, 13], followed by cell regeneration until inflammation is resolved [14]. This negative feedback mechanism enables tissues to dynamically maintain their healthy and youthful state against various external challenges during the entire lifetime. However, most knowledge to construct this theory comes from extrapolation of *in vitro* works and animal injury studies. No human evidence is currently available to demonstrate the role of acute inflammation in the senolytic effect of exercise, as well as its subsequent impact on the pro-inflammatory status in human skeletal muscle.

We recently reported a significant decrease in p16^INK4a^ mRNA levels in the human skeletal muscle of young men, 24 h after HIIE [15]. This senolytic outcome occurred after a significant immune cell infiltration (i.e., CD11b^+^ cells) in muscle tissues immediately post-exercise with elevation in γ-H2AX signals. However, no cell infiltration and senolytic outcomes were observed after moderate-intensity exercise with a similar cycling workload (~70 kJ). These findings suggest that inflammation, typically observed with a higher magnitude of muscle damage, may be necessary to trigger the senolytic effect of exercise in human skeletal muscle. Furthermore, it remains unclear whether the senolytic response can occur as early as within 3 h after high-intensity exercise. The present study was designed to investigate the causality between exercise-induced senolytic effects and inflammation using ibuprofen, a widely used anti-inflammatory drug. While demonstrating efficacy in alleviating pain, the maximum over-the-counter doses of ibuprofen can hinder both strength and muscle hypertrophic adaptations during an exercise training period in young adults [16]. Here we examined the time course of changes in p16^INK4a^ mRNA and pro-inflammatory myeloid differentiation (CD11b mRNA) in human skeletal muscle at 3 h and 24 h following HIIE under ibuprofen-treated and placebo-treated conditions.

## RESULTS

Participant characteristics are shown in Table 1. Rating of perceived exertion (RPE) and maximal heart rate (HR_max_) at the end of HIIE were similar for the placebo and ibuprofen trials.

**Table 1:** Characteristics of participants.

	**Mean ± SE**
Number of participants	12
Age (y)	22.1 ± 0.5
Height (cm)	173.5 ± 1.5
Weight (kg)	74.3 ± 3.5
BMI	23.7 ± 1.0
VO2peak (ml/min/kg)	34.4 ± 2.2
Maximal Aerobic Power (Wmax)	196.5 ± 9.8
Rating of Perceived Exertion (RPE)	
Placebo	6.9 ± 0.5
Ibuprofen	6.9 ± 0.6
Maximal Heart Rate	
Placebo	179.4 ± 4.2
Ibuprofen	178.3 ± 4.3

(Values are represented as mean ± standard error).

**Figure 1:**
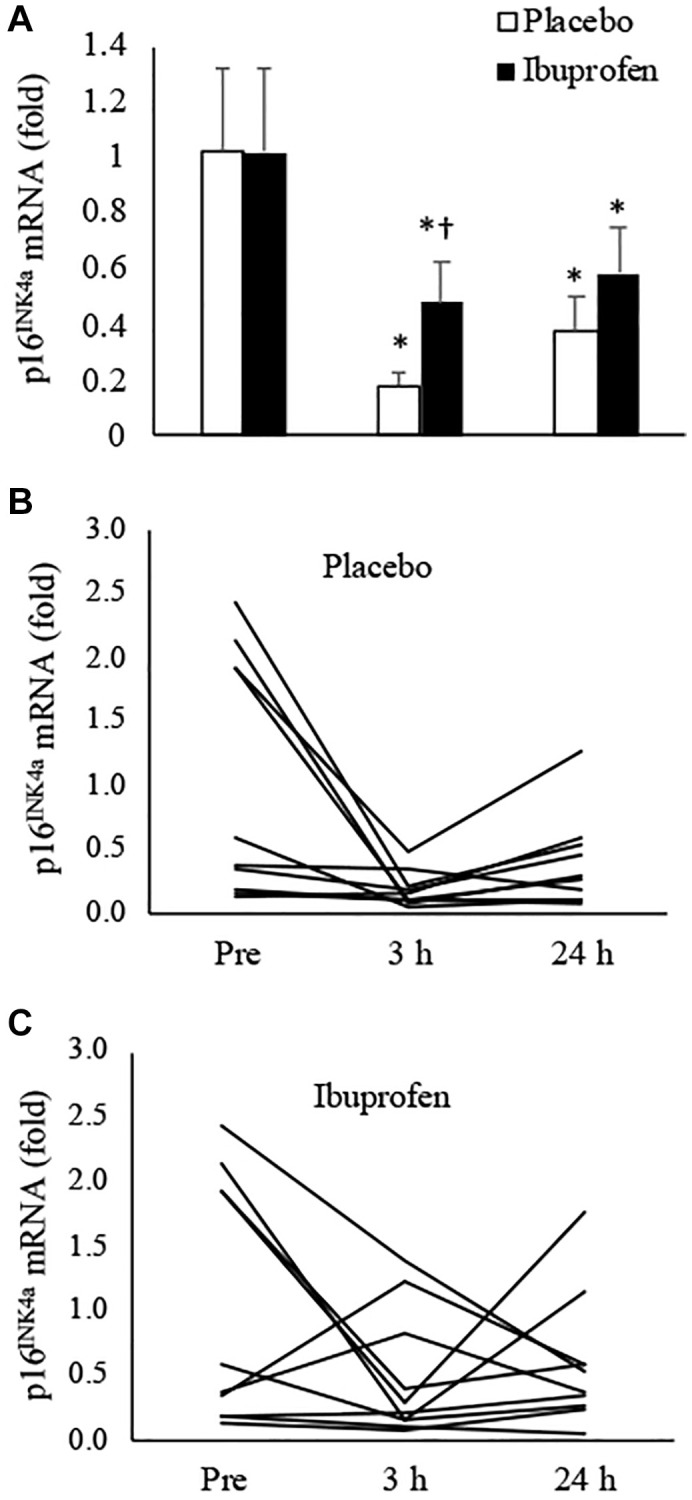
**Effect of ibuprofen on p16^INK4a^ expression in human skeletal muscle after HIIE.** Decreased p16^INK4a^ mRNA after HIIE was attenuated by ibuprofen administration (**A**). A wide individual variation of the exercise response was observed for both placebo (**B**) and ibuprofen (**C**) trials. ^†^Denotes significant difference against placebo, *p* < 0.05; ^*^denotes significant difference against Pre, *p* < 0.05. Abbreviation: HIIE: high intensity interval exercise.

Figure 1 presents the results of p16^INK4a^ mRNA in muscle tissues after HIIE. During the placebo trial, p16^INK4a^ mRNA expression in the skeletal muscle decreased by 82% at 3 h and 62% at 24 h post-exercise, compared to the pre-exercise baseline (Figure 1A) (3 h: *p* < 0.05, *d* = 1.23; 24 h: *p* < 0.05, *d* = 0.88). This response was significantly attenuated by ibuprofen at 3 h after HIIE (placebo vs. ibuprofen: *p* < 0.05, *d* = 2.03), and the difference between groups diminished in 24 h. Individual responses of p16^INK4a^ mRNA in human muscle tissue after placebo and ibuprofen trials are shown in Figure 1B, 1C, respectively. The senolytic effect of HIIE was observed mostly in the muscle samples with high baseline senescence (*n* = 5) before exercise. p16^INK4a^ is exclusively expressed in cells outside myofibers according to immunohistochemical (IHC) analysis.

**Figure 2:**
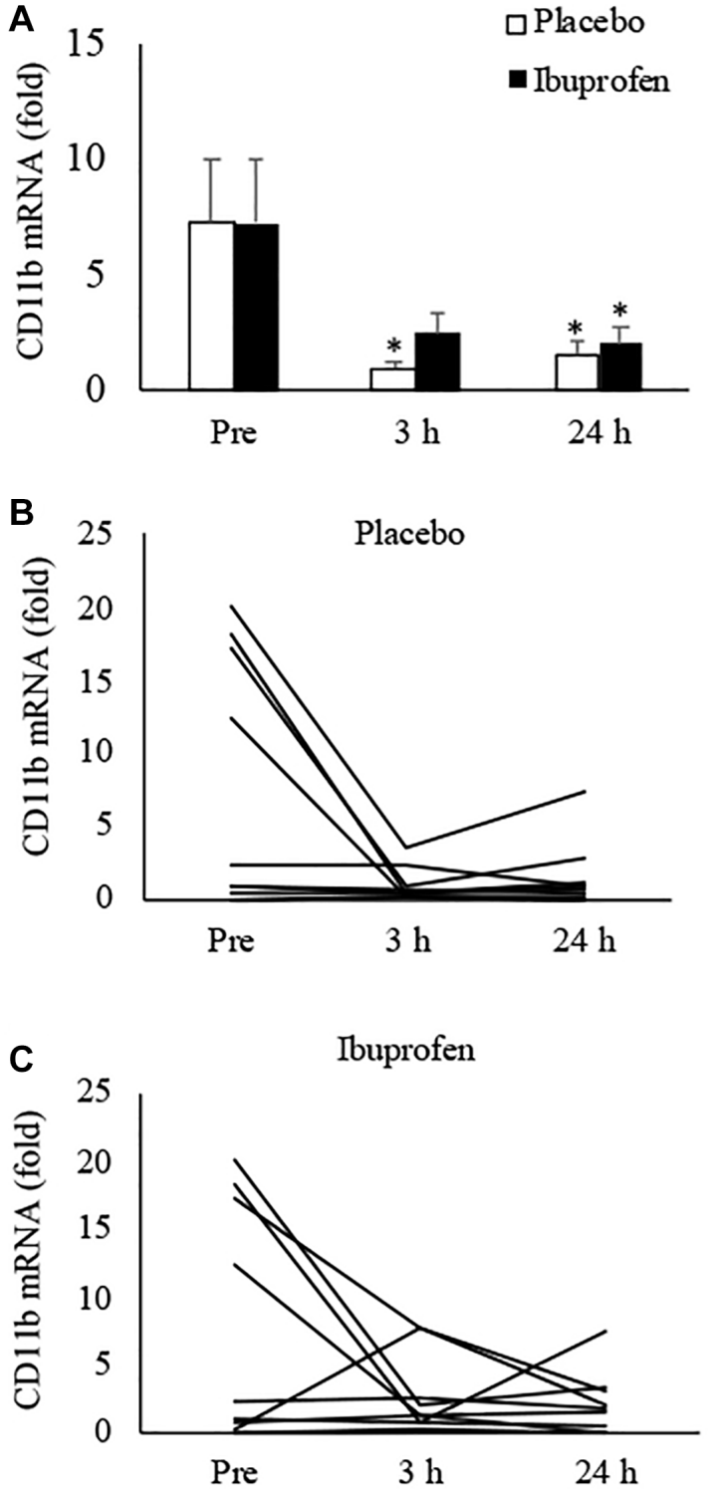
**Effect of ibuprofen on CD11b expression in human skeletal muscle after HIIE.** Decreased CD11b mRNA after HIIE was moderately attenuated by ibuprofen administration (**A**). A wide individual variation of the exercise response was observed for both placebo (**B**) and ibuprofen (**C**) trials. ^†^Denotes significant difference against placebo, *p* < 0.05; ^*^denotes significant difference against Pre, *p* < 0.05. Abbreviation: HIIE: high intensity interval exercise.

**Figure 3:**
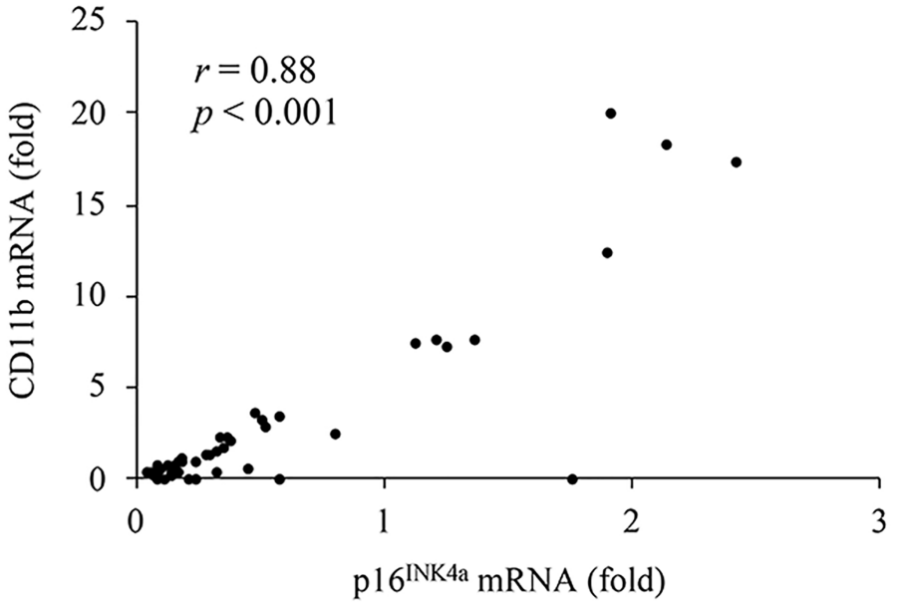
A high correlation between p16^INK4a^ mRNA (cellular senescence) and CD11b mRNA (pro-inflammatory myeloid differentiation) in human muscle tissues.

Figure 2 presents the results of CD11b mRNA expression in human muscle, which indicates pro-inflammatory differentiation of infiltrated myeloid cells [10]. HIIE notably lowered CD11b mRNA in skeletal muscle over the 24-hour recovery period. The decreases reached 87% at 3 h and 80% at 24 h, compared to the pre-exercise baseline (Figure 2A) (3 h: *p* < 0.05, *d* = 1.03; 24 h: *p* < 0.05, *d* = 0.92). This anti-inflammatory effect of HIIE was attenuated by ibuprofen administration, with reductions of only 66% at 3 h and 73% at 24 h post-exercise (3 h: *p* = 0.06, *d* = 0.75; 24 h: *p* < 0.05, *d* = 0.83). The disparity in CD11b mRNA at 3 h post-exercise between the placebo and ibuprofen trials was small (*p* = 0.07, *d* = 0.7). Individual responses of CD11b mRNA in human muscle tissue after placebo and ibuprofen trials are shown in Figure 2B, 2C, which also demonstrate a similar normalization trend of HIIE as seen in the p16^INK4a^ mRNA response. In particular, reductions in CD11b mRNA expression after exercise were observed only in muscle samples with high levels of CD11b mRNA before exercise (3 h: *p* < 0.05, *d* = 2.42; 3 h: *p* < 0.05, *d* = 2.05). A very high correlation between p16^INK4a^ mRNA and CD11b mRNA was observed across all muscle samples in this study (r = 0.88, *p* < 0.001) (Figure 3).

**Figure 4:**
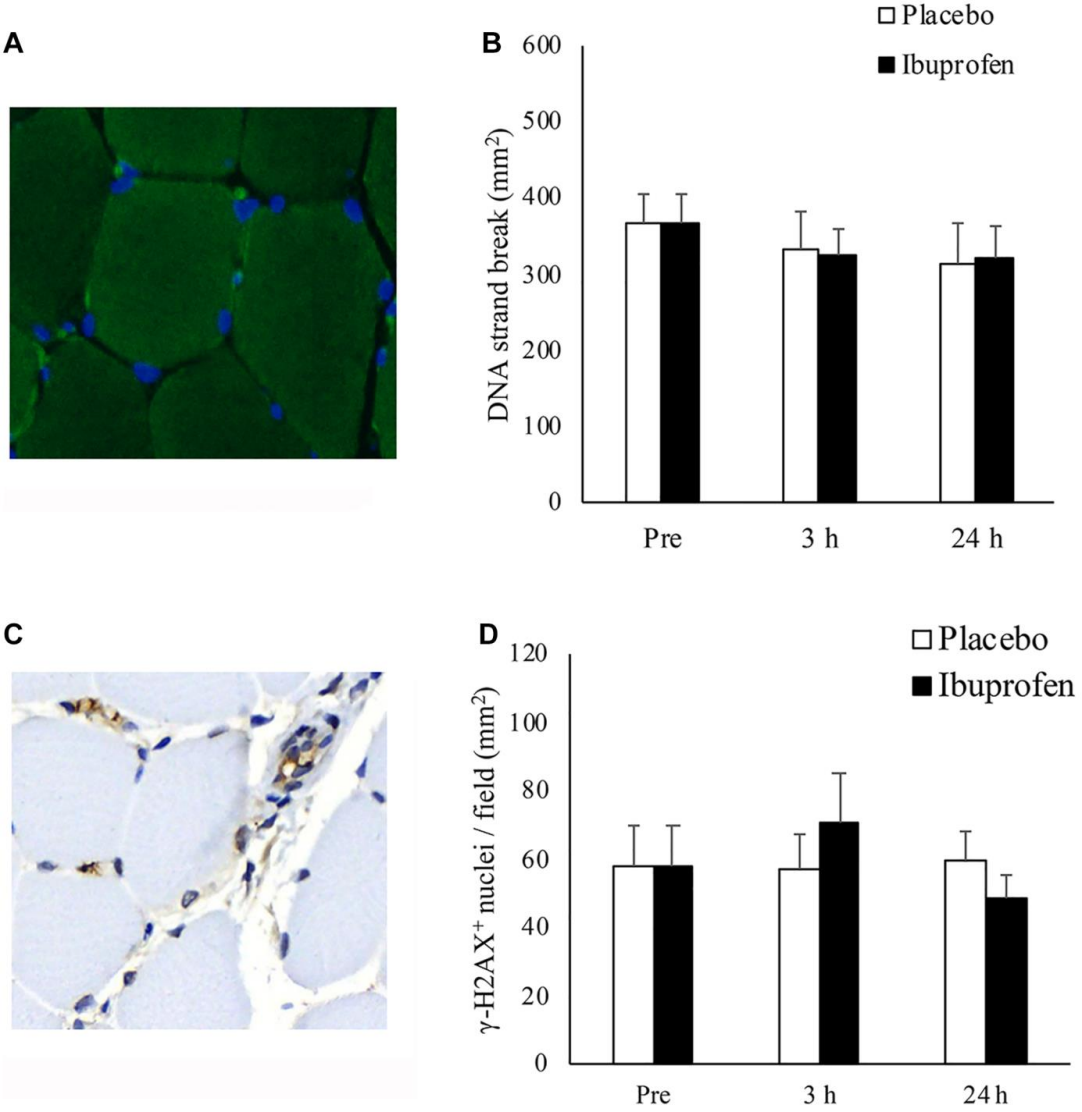
**Effect of ibuprofen on DNA strand break and DNA repair (γ-H2AX^+^ foci) in human skeletal muscle after HIIE.** DNA strand break in nuclei (blue) is visualized by TUNEL immunofluorescence stains (green light) (**A**). DNA strand break at 3 h and 24 h after HIIE was comparable to pre-exercise baseline during both placebo and ibuprofen trials (**B**). γ-H2AX^+^ foci, shown in representative immunohistochemical stains (brown precipitate), are located in both myonuclei and the nuclei outside myofibers (**C**). γ-H2AX^+^ nuclei at 3 h and 24 h after HIIE was similar to pre-exercise baseline during both placebo and ibuprofen trials (**D**). Abbreviations: HIIE: high intensity interval exercise; TUNEL: terminal deoxynucleotidyl transferase dUTP nick end labeling.

Figure 4 illustrates the results of DNA strand break (TUNEL-positive nuclei/mm^2^) and DNA repair markers (γ-H2AX-positive foci/mm^2^) in human skeletal muscle after HIIE. Representative immunofluorescence staining of TUNEL-positive nuclei in biopsied muscle is shown in Figure 4A. Decreases in DNA strand break levels after HIIE from pre-exercise baseline to 3 h and 24 h was small and not statistically significant (Figure 4B). A representative immunohistochemical staining image of γ-H2AX positive foci is shown in Figure 4C. γ-H2AX levels at 3 h and 24 h after HIIE were not significantly different from pre-exercise baseline (Figure 4D).

## DISCUSSION

HIIE is known to induce transient inflammation and DNA damage in skeletal muscle [15]. We have further found that the senolytic effect of exercise in human skeletal muscle occurs only at an intensity high enough to induce significant muscle inflammation (such as HIIE), as opposed to moderate intensity exercises with a similar amount of cycling work [15]. This effect was evident 24 h after exercise. Therefore, in this study we hypothesized that the senolytic effect of exercise is occurred rapidly within 3 h after HIIE and associated with transient inflammation during intense muscle contraction. To test this hypothesis, we measured the senolytic effect of HIIE under an anti-inflammatory condition to determine whether this effect may also occur as early as 3 h after exercise. The key findings of the study are as follows: (1) The most significant reduction in cellular senescence within the muscle occurred within 3 h following an acute session of high-intensity interval exercise (HIIE). Subsequently, the senolytic effect gradually reversed over the next 3 to 24 h; (2) The decreased CD11b expression in immune cells within muscle tissues suggests that a transition from the phagocytic phase to the regenerative phase of inflammation can occur within 3 h following HIIE; (3) Ibuprofen administration delayed both the senolytic and anti-inflammatory effects of HIIE in human skeletal muscle. It is noteworthy that the observed senolytic and anti-inflammatory effects in human muscle were achieved with a very low volume of high-intensity exercise (15 sets of 20-second cycling at 233 ± 11 watts) completed within 10 minutes, and these dual effects persisted for a prolonged period (24 h).

It is generally accepted that p16^INK4a^ mRNA is expressed only in replicable cells [3, 17]. Lowered p16^INK4a^ mRNA levels in skeletal muscle suggests the replenishment of new stem cells in exercised muscle tissues occurs within 3 h after HIIE. In this study, no p16^INK4a+^ signal was detected within myofibers, supporting the notion that the nuclei within myofibers are post-mitotic [18]. The replenishment of damaged nuclei in myofibers is primarily provided by surrounding stem cells [19].

To further examine individual responses among participants, we have found that the significant decrease in p16^INK4a^ mRNA was primarily contributed by the muscle samples with high baseline senescence status. Muscles with low pre-exercise levels of senescence showed negligible changes in p16^INK4a^ mRNA. This *in vivo* evidence suggests that high-intensity exercise provides a stringent selection pressure to stem cells in the late stages of senescence. Furthermore, this outcome indicates that high-intensity exercise can lead to a dynamic shift in the age profile of stem cells residing in human skeletal muscle. Whether this tissue renewal phenomenon is contributed to by Pax7^+^ satellite cell division, CD34^+^ bone marrow stem cell replenishment or decreased phagocytes remains to be examined [20].

In this study, we have found a prolonged reduction in CD11b mRNA 3 h following HIIE, suggesting a reduced proinflammatory myeloid differentiation of cells in skeletal muscle [21, 22]. Integrin CD11b is a surface marker presenting in many types of myeloid cells, particularly in neutrophils [23]. Myeloid cells can differentiate into both phagocytes and muscle stem cells depending on the tissue environment [24, 25]. Phagocytes specifically recognizes and clears senescent cells by phagocytosis, forming a negative feedback mechanism to maintain youthfulness of tissues to lower basal inflammation [12, 13]. The result of the present study, together with our previous findings [15], suggests that a longer period of anti-inflammatory outcomes in skeletal muscle can be attained by a transient pro-inflammatory stimulus from a short episode of HIIE. Ibuprofen is known to inhibit early (phagocytic) phase of inflammation [26], which in turn slow down the entire inflammation program, as the aforementioned negative feedback mechanism for cell renewal induced by exercise. The accumulation of senescent cells in tissues is a key driver of chronic inflammation, marked by a notable increase in proinflammatory mediators [27].

In this study, a very high correlation between p16^INK4a^ mRNA and CD11b mRNA in skeletal muscle, offering compelling *in vivo* human evidence to support this concept. The results also affirm a prior discovery [28], highlighting a significant correlation between the muscle cellular senescence marker p16^INK4a^ mRNA and Myeloperoxidase (MPO) mRNA in human skeletal muscle. MPO, highly expressed in phagocytes, indicates reduced inflammation levels following the clearance of senescent cells after high-intensity exercise.

An acute bout of high intensity exercise can induce an immediate release of bone marrow stem cells into circulation [29]. We have previously shown increased bone-marrow stem cell homing and proliferation in muscle tissues 24 h after HIIE [15, 28]. Chronic inflammation is currently known as a common denominator of age-associated metabolic disorders [30]. Therefore, the prolonged anti-inflammatory response after a brief pro-inflammatory stimulus from HIIE suggests a clinical promise in treating metabolic complications. Despite ibuprofen’s ability to delay senolytic and anti-inflammatory effects following HIIE, the clinical concern is minimal as both placebo and ibuprofen achieved comparably low levels of cellular senescence and decreased CD11b expression after 24 h of recovery.

γ-H2AX^+^ myofibers and fragmented DNA levels in human skeletal muscle elevates immediately after HIIE (0 h), returning to the pre-exercise baseline within 24 h [15]. The speed at which this DNA damage repair can be resolved after HIIE is currently unknown. Our study, adjusted to the biopsy time (3 h after exercise), revealed no significant difference in γ-H2AX levels between the pre-exercise baseline and 3 h post-exercise, indicating a rapid resolution of DNA damage repair following HIIE. γ-H2AX is a protein necessary for recruiting and localizing proteins to form DNA repair machinery [31]. These results align with our earlier findings and with the observation that complete recovery from acute exercise-induced DNA fragmentation can occur within 3 h after moderate prolonged exercise (60 minutes of exercise at 70% VO_2max_) [32]. DNA damage is a factor contributing to cellular senescence, which can increase when the extent of DNA damage exceeds the body’s DNA repair capacity [33].

The present study provides the first evidence demonstrating the importance of acute inflammation on the senolytic effect of exercise. Inflammation is an immune program required for muscle regeneration after injury [14]. Ibuprofen is an anti-inflammatory drug widely used to reduce pain during and after exercise in training athletes. Whether a long-term use of ibuprofen has a negative impact on the positive senolytic effect from exercise remains to be examined. It has been shown that a high dose of ibuprofen (1200 mg/day similar to the present study) attenuated training adaptation in muscle strength and muscle hypertrophy following 8 weeks of resistance exercise in young adults [16]. However, a moderate-dose ibuprofen study showed negligible effect on muscle hypertrophy, muscle strength, and rating of muscle soreness following 6 weeks of resistance training in young adults [34]. It is conceivable that rejuvenated tissues after senescent cell clearance have gained higher capability to extract nutrients compared with the tissues with greater proportion of senescent stem cells. It also remains to be seen whether the same inflammatory-mediating effects occur in the elderly consuming nonsteroidal anti-inflammatory drugs and exercising. For the future, more markers of inflammation and senescence markers such as MPO, SABG, EDU, IL-6 should be measured to demonstrate the inflammatory blockade of ibuprofen.

## CONCLUSION

We provide the first evidence demonstrating an attenuated senolytic effect of HIIE in human skeletal muscle under ibuprofen treatments, suggesting the necessity of inflammation for the exercise-induced senolytic outcomes. Additionally, we further show a diminished pro-inflammatory myeloid differentiation 3 h following HIIE, suggesting a fast transition from the phagocytic phase to the regenerative phase of inflammation.

## MATERIALS AND METHODS

### Participants

Sixteen sedentary young men (aged 20–26 y) who did not engage in regular exercise (less than 1 day per week) were recruited via a website advertisement. Four initially shortlisted applicants withdrew from the study, leaving 12 participants for assessment. They had a range of weights (57–90 kg), heights (171–183 cm), BMIs (20.4–29.4 kg/m^2^), and VO_2peak_ levels (25–48 ml/kg/min) (Table 1). To prevent potential confounding factors related to menstruation-induced inflammation, only male participants were recruited for the experiment.

To ensure study validity, all eligible participants were instructed to abstain from substances that could potentially affect inflammation (such as anti-inflammatory medications, nutritional supplements, and vaccinations), for at least four weeks before and during the study. Individuals with drug allergies, recent smoking history, musculoskeletal, cardiovascular, respiratory disorders, or any inflammatory conditions were excluded from participation. All participants were instructed to maintain their usual levels of physical activity and dietary habits throughout the experimental period.

This study received approval from the Institutional Review Board of the University of Taipei (IRB-2018-078) and adhered to the principles of the Declaration of Helsinki. All participants were thoroughly briefed on the study’s objectives, experimental procedures, and potential risks. Written consent was obtained from each participant before the commencement of the study.

### Study design

To examine the effect of exercise-induced inflammation on cellular senescence of human skeletal muscle after exercise, we performed in a randomized, double-blind, placebo-controlled, counter-balanced crossover trial of the anti-inflammatory medication ibuprofen or placebo with a washout period >3 weeks between trials. Pre-exercise muscle samples were collected 3 weeks before the intervention began. A familiarization cycling trial to determine maximal aerobic power at peak oxygen consumption rate (VO_2peak_) was performed 3 weeks prior to pre-exercise biopsy. Formal assessment for VO_2peak_ was conducted 2 weeks before pre-exercise biopsy. Following HIIE at 120% VO_2peak_, biopsied muscles were collected 3 h and 24 h on the same position of contralateral leg. Body composition was measured before the exercise. Participants were asked to maintain their normal dietary habit during the study.

### Experimental procedures

#### Ibuprofen administration

The participants were blinded with placebo (0.4 g of cornstarch) or ibuprofen (400 mg) capsule (Catalent, Victoria, Australia). The capsule was orally ingested 2 hours before HIIE as well as 3 h and 8 hours after HIIE. The total ibuprofen dosage used in this study was 1200 mg per day. The timing of drug intake is in accordance with the pharmacokinetics of ibuprofen [35].

### VO_2peak_

VO_2peak_ was measured on a cycle ergometer (Monark LC6, Stockholm, Sweden) by using a VO_2_ gas analyzer (Cortex Metalyzer 3B, Leipzig, Germany) to obtain maximal aerobic power (W_max_) at maximal oxygen consumption. Initial pedaling work rate started from 40 watts and the intensity was increased by 20 watts every 2.5 min until volitional exhaustion. If body weight was >65 kg, the initial workload started from 50 watts and followed the same increments. Maximum aerobic power (W_max_) was obtained when VO_2peak_ was reached. VO_2peak_ is defined as the average VO_2_ value during the final 30 sec when a plateau is achieved, marked by an upswing in ventilation during maximal exertion. The value of VO_2peak_ was considered when at least two of four following criteria were achieved: pedaling rate decreased <60 rpm for more than 10 s, respiratory exchange ratio ≥1.2, rating of perceived exertion >9; heart rate ≥100% of age-estimated value.

### HIIE protocol

The exercise protocol was according to our previous published work [15]. In brief, the work rate of exercise challenge was controlled by the Monark Test Software (Monark LC6, Stockholm, Sweden). HIIE started immediately after a 3-min warm-up at 50 watts. Participants cycled at an intensity of 120% W_max_ at 90 rpm for 20 s with a stationary rest interval of 20 s. A total of 15 sets was completed, taking around 10 min. Participants took approximately 3–5 s to reach 90 rpm from the resting condition for each interval. Average cycling power of the participants for each HIIE session was 233 ± 11 watts.

### Rating of perceived exertion (RPE)

Modification of rating of the perceived exertion (RPE) which scaled from 0 to 10 [36] was used in this study to monitor the difficulty to sustain the challenge and was self-reported 5 min post-exercise.

### Muscle biopsy

Muscle biopsies were conducted by an experienced physician using an 18-G Temno disposable cutting needle (Cardinal Health, McGaw Park, IL, USA) inserted 3 cm in depth below skin surface of vastus lateralis, ~20 cm proximal to kneecap. The procedure was conducted in accordance with a previous study [28]. Local anesthesia (2% lidocaine hydrochloride) was administered before the muscle biopsy. The pre-exercise baseline biopsy was conducted on the left leg 3 weeks before HIIE. The second muscle biopsy was conducted on the left leg at the same position 3 h following HIIE. To avoid the acute inflammation induced by the biopsy, the third biopsy was conducted on the contralateral leg at the same position 24 h following HIIE. Muscle tissues were placed on a folded paper for alignment before cross-sectional cutting and immediately kept in a glass vial containing 4% paraformaldehyde solution. Muscle cross-section of the paraffin wax-embedded tissues was used for IHC staining analysis. Part of each muscle sample was immediately kept in liquid nitrogen for real-time PCR analysis.

### Hematoxylin and eosin (HE) staining and immunohistochemistry (IHC) staining

Both HE staining and IHC staining of muscle cross-sections were performed by pathologists from Toson Technology Corporation (Zhubei City, Hsinchu, Taiwan). Leukocyte infiltration in muscle tissue was assessed on HE stained images (TA01HE, BioTnA, Kaohsiung, Taiwan). Muscle tissues were sliced (3 μm thick) from the paraffinized blocks, de-paraffinized in xylene and rehydrated in a graded alcohol series using ethanol (99.9%, 95%, 85% and 75%) each for 2 min, and stained with hematoxylin (TA01NB, BioTnA, Kaohsiung, Taiwan) for 3 min. After being washed in distilled water, muscle tissue sections were stained with eosin for 15 s and dehydrated in ethanol.

Muscle tissue for IHC staining were sliced into cross-sections (3 μm thick) from the paraffinized blocks, de-paraffinized in xylene and rehydrated in a graded alcohol series using 99.9% ethanol, 90% ethanol, 80% ethanol and 70% ethanol each for 4 min before the primary antibody incubation. Samples were then incubated in a de-peroxidase (H_2_O_2_) solution for 5 min at room temperature. Primary antibodies p16^INK4a^ (1:500, #ab108349, Abcam, Cambridge, MA, USA), γ-H2AX (1:200, #ab2893, Abcam, Cambridge, MA, USA), and CD11b (1:200, #ab52478, Abcam, Cambridge, MA, USA) were applied on the slide and incubated for 1 hour at room temperature followed by an incubation to horseradish peroxidase (HRP) conjugated anti-rabbit secondary antibody (TAHC02D, BioTnA, Kaohsiung, Taiwan) labeling for 30 min at room temperature according to the manufacturer’s procedure (BioTnA, Kaohsiung, Taiwan).

To obtain unbiased IHC counting value, two independent assessors counted positive signals of the same image using the same criteria with a minimal acceptable correlation of 0.7. Whole-slide images were viewed using DSAssistant and EasyScanner software at Toson Technology Corporation (Zhubei City, Hsinchu, Taiwan). All glass slides were digitized using multi-level panoramic scanning of pathological slices with a Motic Easyscan Digital Slide Scanner (Motic Hong Kong Limited, Hong Kong, China) at 40X (0.26 μm/pixel). Positive markers within cells were quantified and expressed as positive signal number per total area of skeletal muscle fiber (mm^2^). Only the tissues areas of clustered myofibers were counted. The muscle with disrupted area >40% of muscle cross section was excluded.

### Terminal deoxynucleotidyl transferase dUTP nick end labeling (TUNEL) staining

DNA strand break was assessed with an ApopTag Plus Fluorescein *In Situ* Apoptosis Detection Kit (S7111, Chemicon, Darmstadt, Germany). Muscle tissue sections were fixed in 1% paraformaldehyde and washed in PBS, incubated in equilibration buffer for 10 min and incubated with terminal deoxynucleotidyl transferase (TdT) for 1 h. TdT is a DNA polymerase with good binding specificity to the 3′-OH of oligodeoxyribonucleotides, and commonly used to label fragmentated single-stranded and double-stranded DNA. Muscle tissue sections were then incubated with anti-digoxigenin conjugate for 30 min then washed with a stop buffer. After the PBS wash, muscle tissue sections were mounted with DAPI 4’–6’ diamino-2-phentlindole (DAPI) (Chemicon, Darmstadt, Germany) and TUNEL-positive signals and DAPI-nuclei staining were captured under a fluorescent microscope (ZEISS Axioscan 7, Germany). Fluorescent whole-slide images were viewed and analyzed using Halo Image Analysis Platform software at Taiwan Mouse Clinic-National Comprehensive Mouse Phenotyping and Drug Testing Center. Only TUNEL-positive nuclei overlapping myofiber nuclei were quantified as nuclei with DNA strand break.

### Quantitative polymerase chain reaction (qPCR)

The quantitative PCR followed a standard procedure according to a previous study protocol [28]. RNA was extracted from biopsied muscle (<30 mg) using RNeasy kit (74104, QIAGEN, Hilden, Germany), RNase-Free Dnase Set (79254, QIAGEN, Hilden, Germany), and Proteinase K (19131, QIAGEN, Hilden, Germany) according to manufacturer’s instruction. A total volume of 20 μl was reversely transcribed to cDNA using iScript™ cDNA Synthesis Kit (1708891, Bio-Rad, Hercules, CA, USA). Real-time PCR was performed using MyiQ Single-Color Real-Time PCR Detection System (Bio-Rad, Hercules, CA, USA) and iTaq™ Universal Probes Supermix (1725132, Bio-Rad, Hercules, CA, USA). Gene expression was normalized to the mean of a housekeeping gene (RPP30) of each sample and expressed as a fold change relative to RPP30. The primers and probes for gene expression were supplied from Bio-Rad PrimePCR™ Probe Assay: p16^INK4a^ (or CDKN2A) (Assay ID: qHsaCEP0057827, Bio-Rad, Hercules, CA, USA), CD11b (or ITGAM) (Assay ID: qHsaCIP0025989, Bio-Rad, Hercules, CA, USA), and RPP30 (Assay ID: qHsaCEP0052683, Bio-Rad, Hercules, CA, USA).

### Statistical analysis

Statistical analysis was conducted using IBM SPSS Statistics software, version 27.0. The Shapiro–Wilk test assessed normal distribution, and the Levene test confirmed homogeneity of variances. Comparisons between paired data utilized the nonparametric Wilcoxon signed-rank test. To analyze temporal changes, a Friedman test was applied, and upon detecting a significant F ratio, post hoc analysis was conducted using the Mann–Whitney *U*-test. The significance level was set at *p* < 0.05, and all values are presented as means ± standard error (SE). Cohen’s *d* was used to indicate the effect size of interventions where *d* = 0.2 was considered a small effect size; *d* = 0.5 a medium effect size; *d* = 0.8 a large effect size; and *d* > 1.2 a very large effect size. The probability of type 1 error of 5% was considered significant; with 5–10% considered moderately significant.
